# Supplementary material for: Causal Relationship between Aging and Anorexia Nervosa: A White-Matter-Microstructure-Mediated Mendelian Randomization Analysis
Source: Biomedicines. 2024 Aug 16;12(8):1874. doi: 10.3390/biomedicines12081874 (PMC11351342; doi:10.3390/biomedicines12081874)
Supplement: Supplementary file 1 [file biomedicines-12-01874-s001.zip › Supplementary Figure/Figure S9/Leaveoneout plot of MR analysis for ubm-b-2047 to AN.pdf]

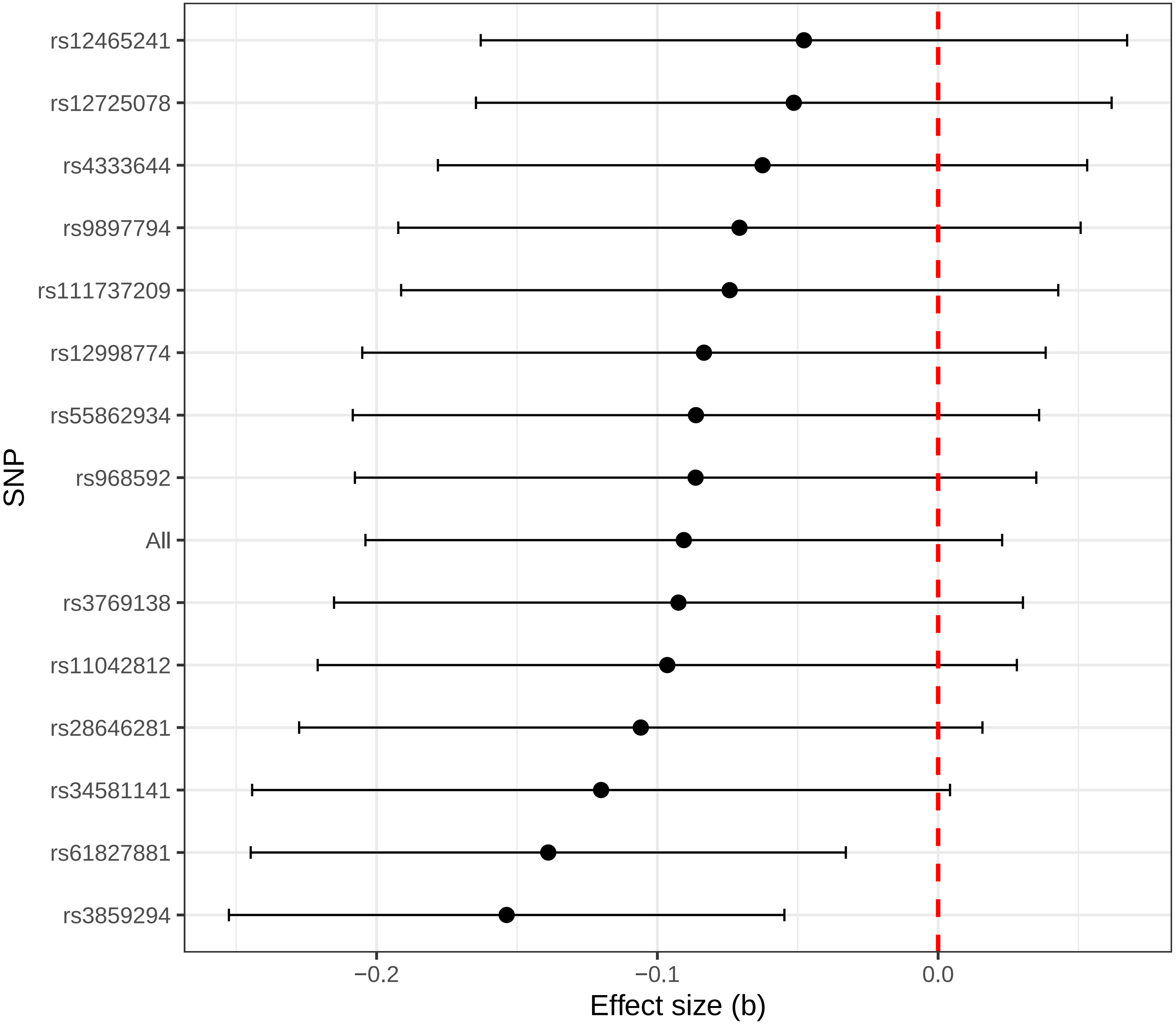
SNP
rs12465241
rs12725078
rs4333644
rs9897794
rs111737209
rs12998774
rs55862934
rs968592
All
rs3769138
rs11042812
rs28646281
rs34581141
rs61827881
rs3859294
−0.2
−0.1
0.0
Effect size (b)
